# Proangiogenic Compositions of Microvesicles Derived from Human Umbilical Cord Mesenchymal Stem Cells

**DOI:** 10.1371/journal.pone.0115316

**Published:** 2014-12-16

**Authors:** Jianying Chen, Zhenjun Liu, Mian Ming Hong, Hongzhe Zhang, Can Chen, Mengyuan Xiao, Junxian Wang, Feng Yao, Mingchuan Ba, Jinghu Liu, Zi-Kuan Guo, Jixin Zhong

**Affiliations:** 1 Division of Cardiovascular Disease, Department of Internal Medicine, Affiliated Hospital of Guangdong Medical College, Zhanjiang, Guangdong, China; 2 Department of Experimental Hematology, Beijing Institute of Radiation Medicine, Beijing, China; 3 Division of Cardiovascular Medicine, Department of Medicine, University of Maryland School of Medicine, Baltimore, Maryland, United States of America; University of Torino, Italy

## Abstract

**Introduction & Objective:**

Microvesicles (MVs) derived from mesenchymal stem cells (MSCs) have been shown to promote angiogenesis. This study was aimed to shed a light on the mechanisms by analyzing the angiogenesis-promoting compositions of MSC-MVs. Also we try to figure out the impact of hypoxia on angiogenesis.

**Methods:**

MVs were isolated from the culture supernatants of MSCs under hypoxia/normoxia and serum-deprivation condition. The morphological features of MVs were revealed by an electron microscope and the origin of the MVs was identified by a bead-bound assay. An antibody array was used to analyze the expression of angiogenic cytokines from MVs and the parent MSCs as well. The major candidate factors were screened and the results were validated by immune blotting.

**Results:**

MSC-MVs were around 80 nm in diameter. They expressed CD29, CD44, and CD73, but not CD31 and CD45. Antibody array showed that both MSCs and MVs expressed many angiogenesis-promoting biomolecules, including interleukin-6 (IL-6), basic fibroblast growth factors (bFGF), and recptor of urokinase-type plasminogen activator (UPAR). MSC-MVs contained angiogenin, vascular endothelial growth factor (VEGF), monocyte chemotactic protein-1 (MCP-1) and the receptor-2 for vascular endothelial growth factor at higher levels than the parent MSCs. Under hypoxic condition most cytokines were expressed in greater quantity than normoxic in MSCs while in MVs there was no significant difference between hypoxic and normoxic conditions except UPAR, Angiogenin, VEGF, IGF, Tie-2/TEK, and IL-6 which were higher in MVs under hypoxic conditions than those in normoxic condition.

**Conclusion:**

Upon serum-deprivation condition, MSCs could secrete MVs that contain a variety of factors contributing to their angiogenesis-promoting function. And among them, Angiogenin, VEGF, MCP-1, VEGF R2 might be of greater importance than the other cytokines. Also UPAR, Angiogenin, VEGF, IGF, Tie-2/TEK, IL-6 might be responsible for hypoxia-augmented proangiogenic effects of MVs.

## Introduction

Mesenchymal stem cells (MSCs), due to their ease of ex vivo expansion, capacity to promote angiogenesis, and the promising pre-clinical data, have been suggested as a possible therapeutic strategy for ischemic disease [1]–[3]. Inefficient MSCs engraftment and differentiation suggest that MSCs act via a secretion-based paracrine effect rather than cell replacement [4]. Culture medium conditioned by MSCs reduced infarct size in animal models of myocardial ischemia/reperfusion (MI/R) [5]. However, the nature of factors responsible for beneficial paracrine effects of MSCs remains elusive.

Many different cell types including MSCs generate microvesicles (MVs) upon activation or apoptosis. MVs are mobile and small vesicles (100–1000 mm) surrounded by the phospholipid bilayer and released by direct budding and blebbing of the plasma membrane. They frequently expose at their surface phosphatidylserine (PS) and express antigenic profile characteristic of the cell they originate. MV is composed of membrane and internal cargo, including lipids, proteins, DNA, mRNA, and micro-RNA. The functions of MVs are not known, but are believed to be important for intercellular communication [6]. Our previous experiments showed that MVs derived from human umbilical cord MSCs stimulated by hypoxia could be internalized into human umbilical vein endothelial cell (HUVEC) and promoted proliferation, migration and in vitro capillary network formation of HUVEC [7]. In a rat hind-limb ischemia model, MSC-MVs were shown to significantly improve the blood flow recovery [7]. Those results indicate that MVs releasing may be one of the major mechanisms underlying the effectiveness of MSCs therapy by promoting angiogenesis both in vitro and in vivo. MVs-based therapy circumvents some of the concerns and limitations in using MSCs, such as embolism and abnormal differentiation, therefore might be as an alternative to MSCs for curing ischemic diseases. In this article by comparing the level of angiogenic cytokines in MVs and their parent MSCs, we try to figure out the major candidate factors promoting angiogenesis. Also by comparing the expression of angiogenesis cytokines under hypoxia and normoxia, we try to figure out the impact of hypoxia on angiogenesis.

## Materials and Methods

### MSC isolation and culture

Human umbilical cords were harvested after obtaining written informed consent, according to the Ethical Guidelines for Research Involving Human Subjects the Ethical Guidelines for Research Involving Human Subjects or Human Tissue from General Hospital of Air Force. All procedures have been reviewed and approved by the Institutional Review Board (IRB) of Guangdong Medical College. Umbilical cords were cut into small pieces and the arteries were removed. The cells were cultured in alpha minimal essential medium (α-MEM) containing 10% fetal bovine serum (FBS) selected from lots (Stem Cell Co. Canada) and 100 U/ml penicillin/100 µg/ml streptomycin. Cells were maintained at 37°C, 5% CO_2_. Culture medium was changed every 2–3 days. MSCs were passaged at a confluence of 80–90%. MSCs at passage 3–5 were used in all the experiments below.

### MSC differentiation

Osteogenic differentiation was examined by alkaline phosphatase (ALP) staining after cells were cultured in the osteogenic differentiation medium for 14–21 days; medium was changed every 3–4 days. Adipocyte-like cells were identified by Oil Red O staining after cells were cultured in the adipogenic differentiation medium for 14–21 days.

### MSC phenotypic characterization

For flow cytometric analyses MSC were stained with fluorescein isothiocyanate (FITC) or phycoerythrin(PE)-conjugated murine antibodies against human CD14, CD31, CD73, CD44, CD106, CD45, and PDGFR. Mouse isotypic antibodies served as controls. 1×10^6^ cells were suspended in 1 ml PBS containing 10% FBS and aliquots of 100 µl were incubated with labeled antibodies for 30 min at 4°C and then washed twice with PBS. Fluorescence of 10,000 viable cells was analyzed with a flow cytometer (FACS calibur, BD Biosciences USA) and the data were analyzed with WinMDI 2.9 software.

### MVs harvest

Aliquots (1×10^6^) of MSCs were plated into 150 mm-dishes in α-MEM containing 10% FBS. The MVs were eliminated from serum by ultra-centrifugation (100,000 *g*, 1 hour) before its use as described elsewhere [8]–[10]. After 5 washes with 1x PBS and 3 times with serum-free medium, culture medium was replaced with α-MEM deprived of FBS when cells were at a confluence of 75%. Cell culture was maintained under hypoxic condition (1% O_2_) or under normoxia condition for 72 hours. Cells were collected and counted using a haemocytometer following resuspension in 1 ml medium and using trypan blue to exclude dead cells in the total cell count. Cell supernatant was centrifuged at 16,500×g for 20 min to remove dead cells and cell debris, and then filtered through a 0.45 µm Super membrane (Pall Life Sciences, USA) to discard vesicles larger than 450 nm. Supernatant was then transferred to ultracentrifuge tubes (14×89 mm, Beckman, USA) and ultracentrifuged at 100,000×*g* for 60 *min* at 4°C, followed by a second ultracentrifugation in the same conditions to wash MVs from soluble proteins, protein aggregations and other contaminants that might co-pellet with the MVs. The pellet were resuspended in 50 µl apop buffer containing 5 mM KCl, 1 mM MgCl and 136 mM NaCl for one ultracentrifuge tube, split into aliquots of 5 µl and stored at −80°C for the use in the experiments below.

### MVs quantification

The determination of the amount of MVs was carried out by measuring total MVs-associated proteins,using bicinchoninic acid protein assay kit (Applygen Technologies Inc) and was done according to the manufacturer's protocol.

### MVs electron microscopy

A drop of approximately 10 µg of MVs were fixed with with paraformaldehyde to copper mesh formvar grids. Grids were further fixed with 1% glutaraldehyde and negatively stained by 1% Phosphotungstic acid. Sample were observed using scanning electron microscope (Hitachi H-7650, Japan) at a working voltage of 30 kV and images were taken at a magnification of x10,000.

### MVs phenotypic characterization

MVs phenotypic profile was determined by a bead-based flow cytometric technique described below. In brief, 5 µg purified MVs were incubated with 0.1 µl aldehyde/sulfate latex beads (4 µM, Molecular Probes, Invitrogen, USA) for 1 h at room temperature, the total amount of beads is about 10^5^. Add MES buffer (0.025 M MES, 0.154 M NaCl, pH 6.0) to a final volume of 1 ml, and incubate on a test tube rotator wheel overnight, add 110 µl of 1 M glycine (i.e., 100 mM final), mix gently and let stand on the bench at room temperature for 30 min. Wash three times with MES/3%FBS and resuspend the bead pellet in 90 µl MES/3%FBS. Incubate with 10 µl FITC or PE-conjugated murine antibodies against human CD31, CD73, CD44, CD29, and CD45 for 40 min at 4°C and then washed twice. Mouse isotype andibodies were used as control. Analyze antibody-stained MVs-coated beads on a flow cytometer. Single beads and bead doublets were gated and the relative fluorescence was analyzed with WinMdi2.9 software.

### Antibody array

MVs and their parent MSCs harvested under hypoxia and normoxia from three individual experiments were lyzed with 2X Cell Lysis Buffer (RayBiotech, USA) and quantified using a bicinchoninic acid protein assay kit (Applygen Technologies Inc). Lysates were then analyzed for angiogenesis-related cytokines (60 cytokines total) using a RayBio Quantibody Human Angiogenesis Array 1000 kit (RayBiotech, USA). The assay was performed as instructed by the manufacturer. In brief, 100 µl Blocking Buffer was added into each well and incubated at room temperature for 60 min to block slides. After removing blocking buffer, 30 µg of samples or serial diluted cytokine standards were added to each well containing 70 µl Sample Diluent. After overnight incubation at 4°C, decant the samples and wash three times with wash buffer I at room temperature with gentle shaking. Wash twice with wash buffer II. Add 70 µl diluted Detection Antibody to each well. Incubate at room temperature for 2 h and wash as above. Add 80 µl diluted Cy3 equivalent dye-conjugated streptavidin to each well. Incubate in dark room at room temperature for 1 hour and wash as above. Scan the signal of array glass chip by Axon GenePix (GenePix 4000B, Axon Instruments, USA). Data was analyzed using GenePix Pro 6.0.

### Western blot

Lysates of MVs and their parent MSCs harvested under hypoxia or normoxia (50 µg) were electrophoresed through a 12% SDS-polyacrylamide gel. The proteins were then transferred to a PVDF membrane at 200 mA for 2 h, after which Western blot analysis was conducted, After blocking with 5% skim milk, the membrane was subsequently incubated with 1∶400 diluted rabbit anti-UPAR, 1∶200 diluted mouse anti-Angiogenin, or 1∶1000 diluted mouse anti-GAPDH (Santa Cruz Biotechnology, Santa Cruz, USA) and washed three times for 10 min with TBST buffer. Next, the membrane was incubated with HRP conjugated anti-rabbit or anti-mouse IgG. After being washed three times with TBST buffer for 10 min, the HRP activity was observed by applying a chemiluminescent substrate (Applygen Technologies In) and exposing the samples to an X-ray film.

### Statistical analysis

Student's t-tests (for comparisons between two groups) or one-way ANOVA (for comparison of three or more groups) followed by Tukey post hoc tests were used for statistical analyses. A value of p<0.05 was considered statistically significant.

## Results

### MSC characterization

**Figure 1:**
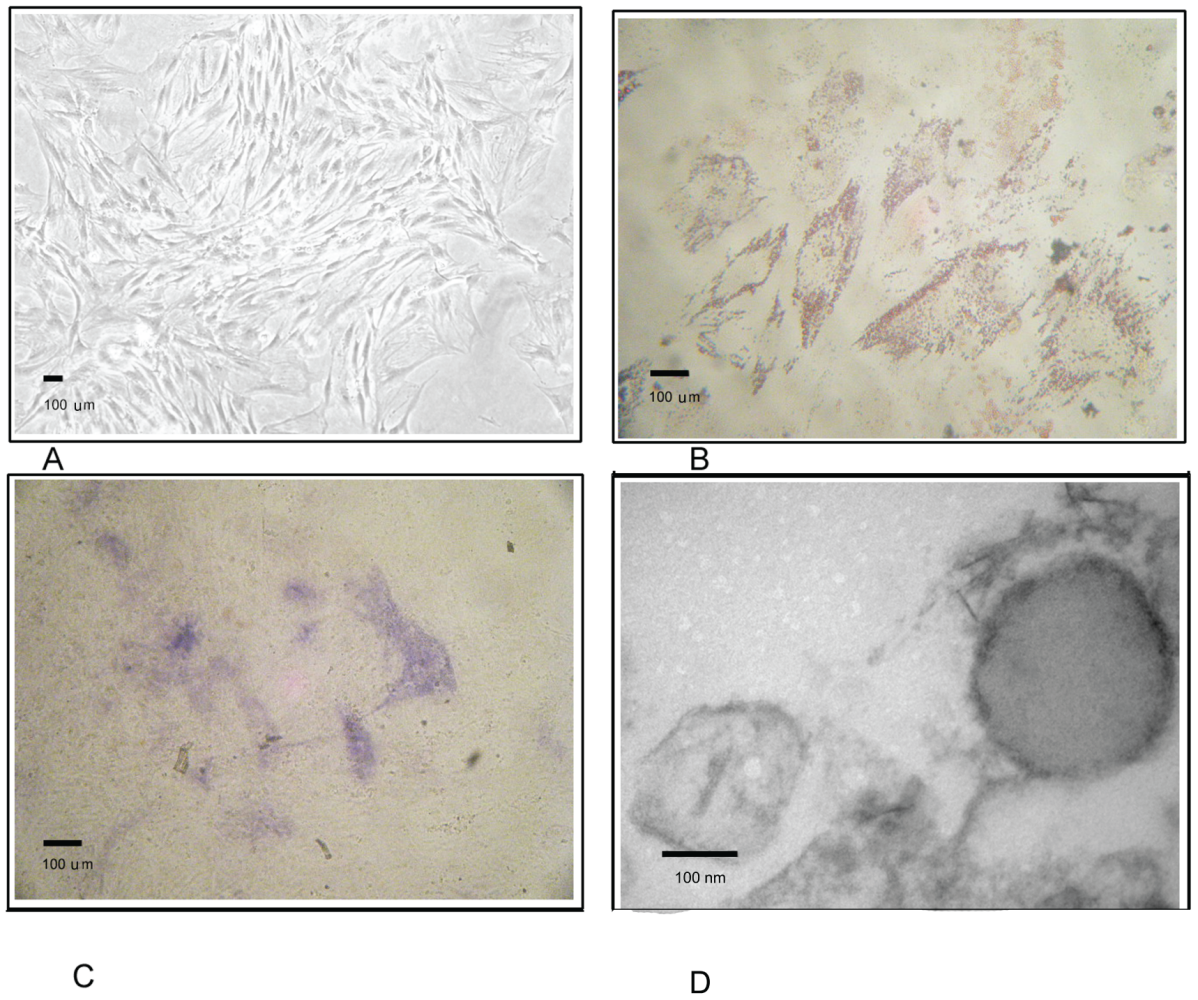
Characteristics of MSCs. A. Umbilical cord derived MSCS. B. Adipogenic differentiation of MSCs was examined by Oil Red O staining. C. Osteogenic differentiation was examined by alkaline phosphatase staining. D. Morphological features of MVs under electron microscope.

**Figure 2:**
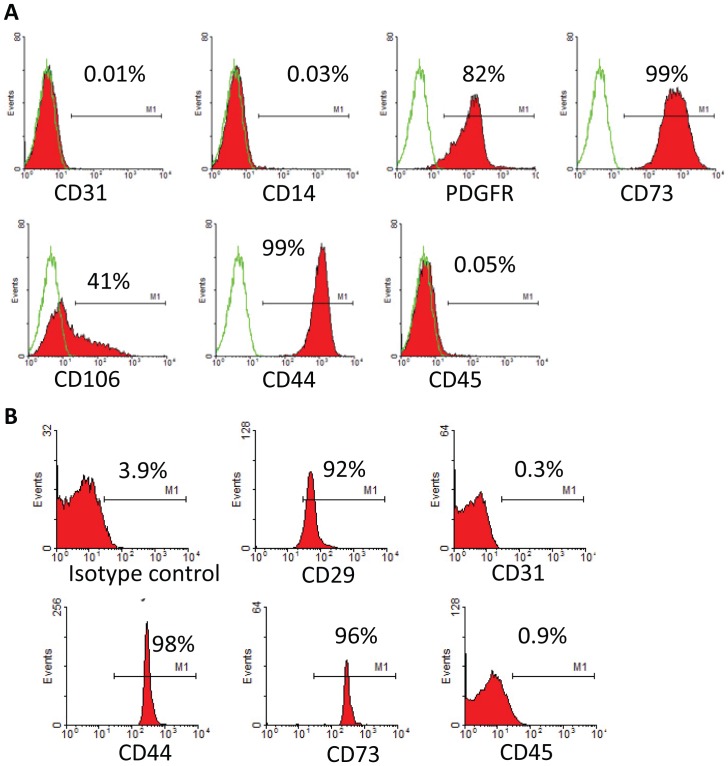
Flow cytometric characterization of MSC and MV. A. Detection of MSC-specific marker expression of UMSC by flow cytometric analysis. B. Identification of MSC-MVs by bead-based flow cytometry.

At 5 days post isolation, the cells showed an elongated, triangle-like shape, with many of them showing mitotic figures. At 10 days the cells reached full confluence and acquired a more pronounced fibroblast-like shape (Fig. 1A). Cells showed multilineage cell differentiation potential into adipogenic, osteogenic under the influence of lineage-specific, differentiation culture media (Fig. 1 B, C). The majority of cells showed a prominent presence of the MSC surface markers CD73, CD44, CD106 as well as PDGFRβ. There was little if any expression detectable of CD31, CD14, and CD45 respectively, suggesting the absence of endothelial and hematopoietic cells types (Fig. 2A). Cultivation of MSC under hypoxia revealed higher proliferation activity of the cells without changes in morphology conditions. Cell count of viable MSCs was 0.97±0.06×10^7^ at day 0 and cell number after 3 days of culture under hypoxia serum-deprived condition was higher than that under normoxia (1.4±0.08×10^7^ vs. 0.86±0.06×10^7^, p<0.05).

### MVs characterization

Under an electron microscope, MSC-MVs took a round morphology, and their diameters were around 100–200 nm. All of which were typical for MVs described elsewhere (Fig. 1D). Bead-based flow cytometric analysis showed that MSC-MVs were positive for CD44, CD73, and CD29 and negative for CD31 and CD45. These results suggested that the MVs were originated from MSCs (Fig. 2B). Three independent experiments showed that total protein content of MVs harvested upon hypoxia condition was 252±40 µg/10^7^ MSCs, significantly higher than upon normoxia (130±25 µg/10^7^ MSCs p<0.05).

### Antibody array

**Figure 3:**
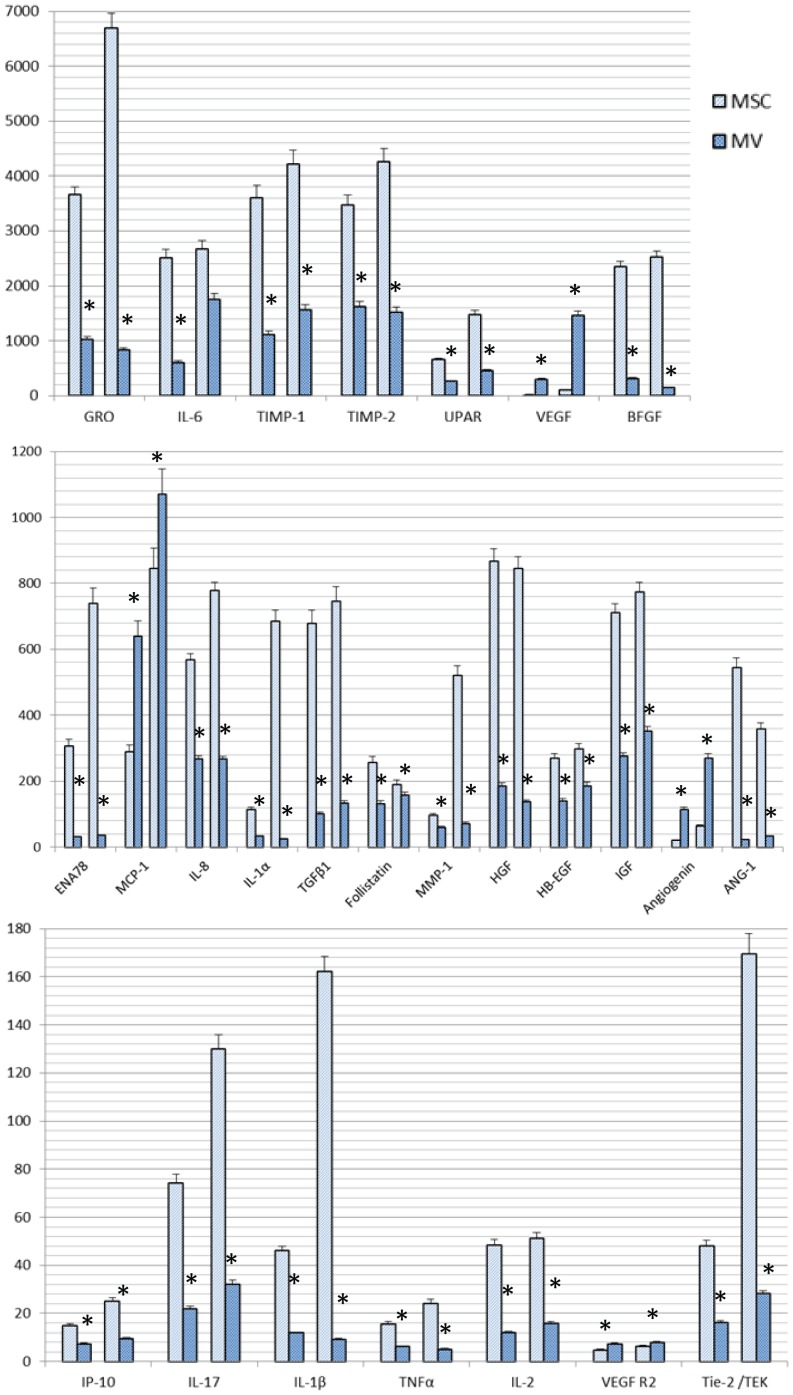
Comparison of cytokine concentrations (pg/ml) in MVs and parent MSCs detected by antibody array. A represents normoxic condition and B represents hypoxic condition. Results are expressed as mean ± SD. *, P<0.05.

**Figure 4:**
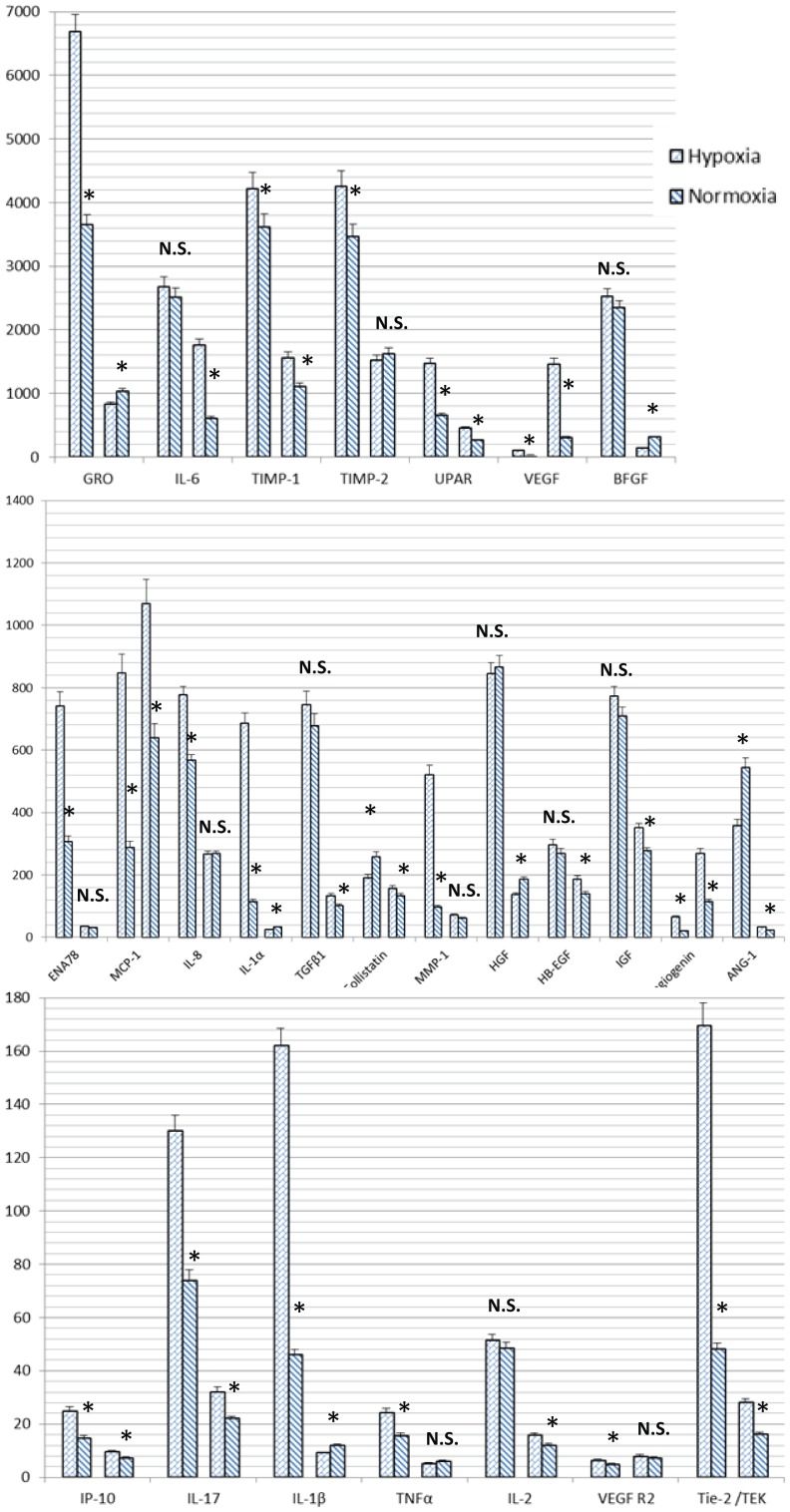
Comparison of cytokine concentrations (pg/ml) under hypoxic and normoxic conditions detected by antibody array in MSCs and MVs. A represents MSCs and B represents MVs. Results are expressed as mean ± SD. *, P<0.05; N.S., P>0.05.

We used Human Angiogenesis Array to analyze the expression of 60 angiogenesis-related cytokines from MVs and their parent MSCs harvested under hypoxia or normoxia. The results showed that uPAR, Metalloproteinase inhibitor 2 (TIMP-2), growth-related oncogene (GRO), Metalloproteinase inhibitor 1 (TIMP-1), Insulin like growth factor I (IGF-I), Basic fibroblast growth factors (bFGF), interleukin-6 (IL-6), interleukin-8 (IL-8), VEGF, Hepatocyte growth factor (HGF), Transforming growth factor β1 (TGFβ1), MCP-1, MMP-1, Tie-2/TEK, ENA-78, IL-1α, Follistatin, Angiogenin, IL-1β, VEGF R2, MCP-3, MCP-2, GM-CSF, ANG-1, HB-EGF, IL-2, IL-17, IP-10, RANTES, TNFα, I-TAC were present in both MVs and MSCs. Among these, uPAR, TIMP-2, GRO, TIMP-1, IGF-I, bFGF, IL-6, IL-8, VEGF, HGF, TGFβ1, MCP-1 were expressed abundantly, all more than 50 pg in 30 µg MSCs total proteins and 10 pg in 30 µg MVs total proteins. While IL-4, IL-10, IL-12p40, IL-12p70, TGFα, TGFβ3, Tie-1, VEGF R3, VEGF-D, Angiostatin, TNFβ, AgRP, Leptin, PDGF-BB, PIGF, CXCL16, PECAM-1, MMP-9, I-309/CCL1, MCP-4 were expressed neither in MSCs nor MVs. We compared the quantity of cytokines expressed in MSCs and MVs and found no matter under hypoxic or normoxic conditions most of cytokines were expressed in greater quantity in MSCs than MVs (p<0.05), except angiogenin, VEGF, MCP-1, VEGF R2 which were expressed greater in MVs than MSCs (Fig. 3). Also we compared the quantity of cytokines expressed under hypoxic or normoxic conditions and found in MSCs most cytokines were expressed in greater quantity under hypoxic than under normoxic condition (*p* = 0.013), while in MVs there was no significant difference between hypoxic and normoxic conditions for most of the cytokines (*p* = 0.171), except UPAR, angiogenin, VEGF, IGF, Tie-2/TEK, IL-6 which were expressed greater under hypoxic than normoxic (Fig. 4).

### Western blot

**Figure 5:**
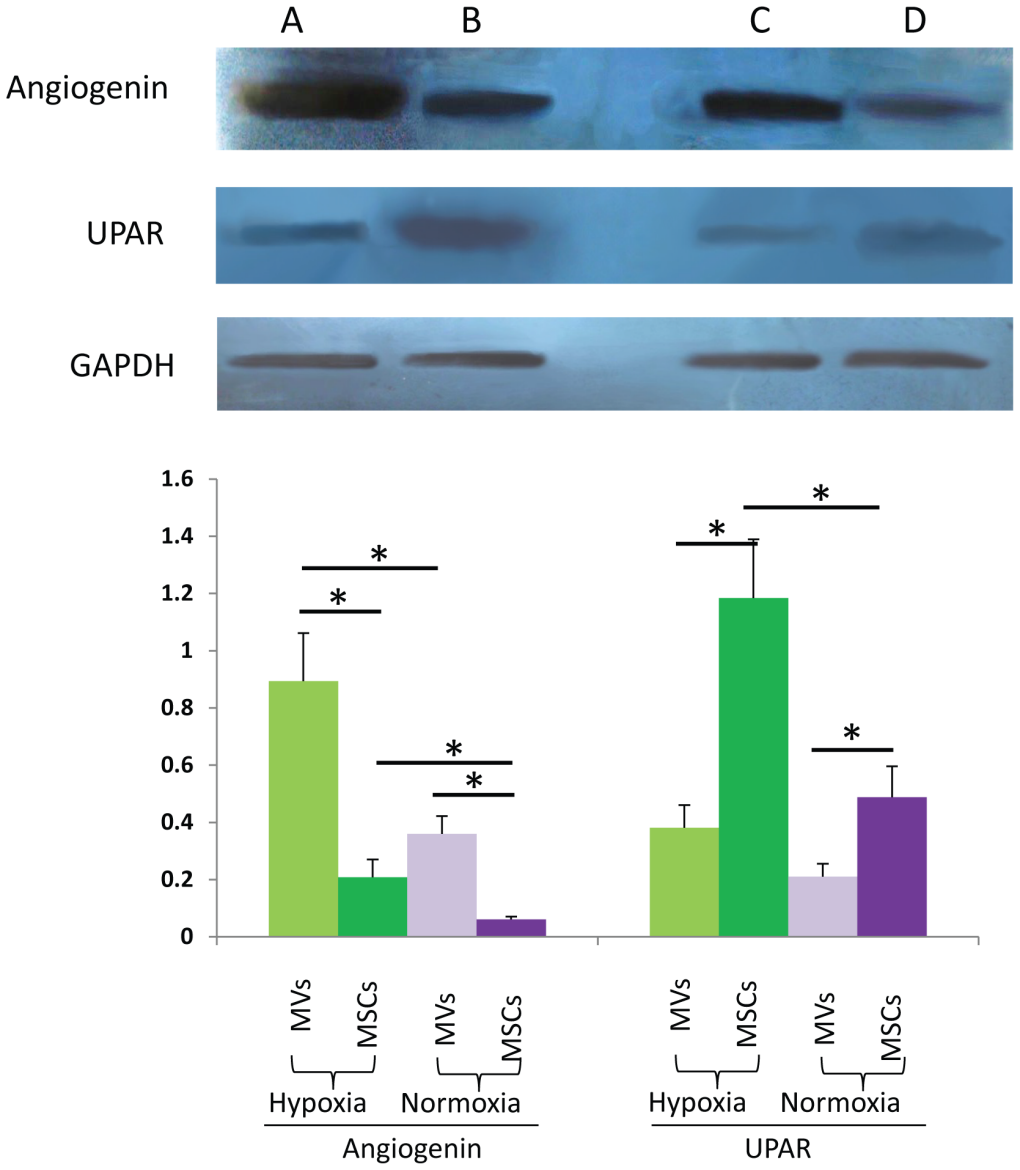
Western blot analysis of angiogenin and UPAR in MVs and MSCs harvested of under hypoxia or normoxia. A. MVs harvested under hypoxia condition; B. parent MSCs under hypoxia condition; C. MVs harvested under normoxia condition; D. parent MSCs under normoxia condition. GAPDH was used as an internal reference. Lower: Relative UPAR (UPAR/GAPDH) and angiogenin (angiogenin/GAPDH) levels; data shown are representatives of at least three independent experiments. * P values <0.05.

Based on the results of antibody array, we selected UPAR and Angiogenin for immunoblotting detection in MVs and parent MSCs to validate the result of antibody array. GAPDH was used as an internal reference. The result of western blot was mainly in accordance with our antibody array (Fig. 5).

## Discussion

It is generally accepted that MVs mediate cell to cell communication by horizontal transferring their internal cargo including lipids, proteins, mRNA and microRNA across plasma membranes and elicit cellular responses. MVs are known to bear numerous membrane proteins that have binding affinity to other ligands on cell membranes or extracellular matrix; these membrane bound molecules provide a potential mechanism for cellular uptake of MVs by specific cell types. Many of the proteins in MVs are enzymes, the effect of enzyme-driven biological activities are catalytic and not stoichiometric and could be activated or attenuated in proportion to the severity of disease-precipitating microenvironment [11]. In contrast to RNA, protein in MVs could mediate a more rapid “off-the-shelf” therapeutic response to repair. Therefore our experiment focused on the protein of MVs relating angiogenesis by using highly sensitive antibody-based techniques which enable the detection of very low abundant yet highly potent growth factors and cytokines.

Our results showed that the following cytokines were expressed in both MVs and MSCs. These cytokines can be classified into the following groups: Chemokine's: MCP-1 (CCL2), RANTES (CCL5), MCP-3 (CCL7), MCP-2 (CCL8), GRO (CXCL1), ENA-78 (CXCL5), IL-8 (CXCL8), IP10 (CXCL10), I-TAC (CXCL11); Pleiotropic cytokines: IL- 1β, IL-6; Pro-inflammatory cytokines: IL-1α, IL-2, IL-17, TNFα; Anti-inflammatory cytokines: TGFβ1, Follistatin; Growth and trophic factors: Granulocyte-macrophage colony-stimulating factor (GM-CSF), IGF-I, BFGF, HGF, Human Proheparin-Binding Epidermal growth factor (HB-EGF); Angiogenic factors: VEGF, Angiogenin, Angiopoietin-1 (ANG-1); Receptors: uPAR, Tie-2/TEK, VEGF R2; Proteases and inhibitors: Matrix metalloproteinase 1 (MMP1), TIMP-1, TIMP-2.

Angiogenesis is the growth and proliferation of new capillaries from an existing vascular structure. Therapeutic angiogenesis is an approach to stimulate angiogenesis to improve perfusion, deliver survival factors to sites of tissue repair, and ultimately restore form and function to the tissue. Angiogenesis is a complex biological process comprising many different steps that are precisely regulated. The cytokines above may play a role in these steps: First, vasodilatation of the pre-existing vessel and formation of vesiculo-vacuolar organelles in the endothelial cells. The most important effector for this is vascular endothelial growth factor (VEGF) [12]. Second, vessel destabilization and matrix degradation, angiopoietin and proteases are involved in this step [13]. Two isoforms of angiopoietins, Ang1 and Ang2, and their tyrosine kinase receptors (Tie1, Tie2) regulate sprouting angiogenesis, vascular remodeling and endothelial cell activation [14]. TIMP-1 and TIMP-2 are endogenous inhibitors of MMPs. Small amounts of MMPs are essential for the onset of morphogenetic program, while excess amounts prevent tubulogenesis. TIMP maintains a delicate balance between proteases and their inhibitors [15]. UPAR acts by transferring to endothelial cell and bind with endogenous U-PA to activate plasminogen into plasmin, exerting its proteases activities [16]. Third, endothelial cell proliferation and migration. Specific mitogens of endothelial cells in this step are VEGF and angiopoietins, as well as other molecules, such as angiogenin and FGF, epidermal growth factor, CXC chemokines, and IGF-I. Fourth, lumen formation and vessel stabilization, different forms of VEGF, integrins and TGFβ1 have been shown to be implicated in this step [17].

UPAR, TIMP-2, GRO, TIMP-1, IGF-I, bFGF, IL-6, IL-8, VEGF, HGF, TGFβ1, MCP-1 were expressed abundantly in both MVs and parent MSCs, with a level of more than 50 pg in 30 µg MSCs total proteins and 10 pg in 30 µg MVs total proteins. These cytokines might be candidate angiogenic factors for MVs [12]–[17]. While IL-4, IL-10, IL-12p40, IL-12p70, TGFα, TGFβ3, Tie-1, VEGF R3, VEGF-D, Angiostatin, TNFβ, AgRP, Leptin, PDGF-BB, PIGF, CXCL16, PECAM-1, MMP-9, I-309/CCL1, and MCP-4 were not expressed in either MSCs or MVs, suggesting that these factors are less important for MVs to promote angiogenesis. The information about proteins, RNA, and lipids identified up to date in MVs is collected in VESICLEPEDIA, a manually curated web based database (http://www.Microvesicles.org). We retrieved VESICLEPEDIA and confirmed that Angiogenin, UPAR, IL8, MMP1, TIMP1, TIMP2, MCP-3, HGF, and TGFβ1 have been identified as MSC-deriving MVs protein, which is consistent with our result.

Liu et al [18], analyze the expression of cytokines from culture medium of MSCs using a cytokine protein array. Several cytokines including ENA-78, GM-CSF, GRO, IL-1b, IL-6, IL-8, MCP-1, VEGF, FGF-4, TGF-β2, TIMP-1, as well as TIMP-2, were detected. IL-6, IL-8, TIMP-1, and TIMP-2 were the most abundant interleukins expressed by MSCs. Their result of culture medium is in part consistent with ours. The proteomics analysis of the secretome by Jung et al [19], revealed that 40% of proteins were classified into cytosolic proteins, many of which were MVs-associated proteins. Taken together, we postulate that many of the secreted proteins were encapsulated in MVs.

We compared the quantity of cytokines expressed in MVs and parent MSCs and found no matter under hypoxic or normoxic conditions most cytokines were expressed in greater quantity in MSCs than MVs, except Angiogenin, VEGF, MCP-1, and VEGF R2 which were expressed at a higher level in MVs. As we mentioned above, the sorting and encapsulating of functional proteins from MSCs into MVs is a very precise and intelligent process. We postulate that Angiogenin, VEGF, MCP-1, VEGF R2 are selectively transferred to MVs from the mother MSCs in a great quantity while the other angiogenesis cytokines just transferred seldom. Accumulating evidence suggest that the angiogenesis-promoting effects of MSCs are predominantly caused by secretion-based paracrine and those cytokines which are secreted seldom could not play a crucial role, indicating that Angiogenin, VEGF, MCP-1, and VEGF R2 might be of greater importance for MVs to promte angiogenes. VEGF, also known as vascular permeability factor (VPF), is a potent endothelial cells specific mitogen and permeability-enhancing factor that has been shown to play a central role in angiogenesis. Activation of VEGFR-2 by VEGF is a critical requirement to induce the full spectrum of VEGF responses. VEGF promotes endothelial cells survival, proliferation and migration through numerous pathways, including activation of the MAPK, extracellular signal-regulated kinase (ERK), p38 and c-jun N-terminal inase (JNK), and Rho-GTPase family members [20]. MCP-1 belongs to CXC chemokine family members, and is able to affect endothelial cells migration, promote angiogenesis and reduce apoptosis by reducing caspase-3 activity [21]. Angiogenin is a 14 Da soluble protein and a member of the ribonuclease (RNase) superfamily. Angiogenin induces angiogenesis by activating vessel endothelial and smooth muscle cells and triggering a number of biological processes including cell migration, invasion, proliferation, and formation of tubular structures. It has been reported that angiogenin plays its functions through exerting its ribonucleolytic activity RNase activity directing towards 28S and 18S rRNA or binding to membrane actin and then inducing basement membrane degradation [22].

We compared cell count of MSCs after cultured for 3 days under hypoxia or normoxia condition, results showed cultivation under hypoxia revealed a higher proliferation activity than normoxia. We compared the total protein content of MVs harvested upon hypoxia and normoxia condition. Result showed hypoxia induces MSCs to produce more MVs than normoxia. Hypoxia and serum deprivation might be efficient ways for MVs preparing. Hu et al. [23], reported hypoxic preconditioning enhances the capacity of MSCs to repair infarcted myocardium, attributable to increased angiogenesis. We compared the quantity of cytokines expressed under hypoxic and normoxic conditions and results showed most of cytokines in MSCs were expressed in greater quantity under hypoxic than under normoxic condition. MSCs were induced to express hypoxia-inducible factors (HIFs) upon hypoxia stimulation. The induction of HIF expression in MSCs might enhance their proangiogenic activity by secreting a variety of growth factors (VEGF, FGF2, and HGF) in favor of survival and enhance the expression of anti-apoptotic Bcl-2 [24]. However, in MVs there was no a significant difference between hypoxic and normoxic conditions for most of the cytokines, except UPAR, angiogenin, VEGF, IGF, Tie-2/TEK, IL-6. Paulina et al. [25], indicating that MVs derived from hypoxic cells are more potent inducers of angiogenesis ex vivo and in vitro. Salomon et al. have investigated the impact of hypoxia on MVs release from MSCs and their pro-angiogenic activity [26]. They found in response to hypoxic conditions (1% and 3% O_2_), MVs release increased by 3.3- and 6.7-fold respectively. MVs derived from MSCs increased placental microvascular endothelial cells (hPMEC) migration by 1.6-fold and increased hPMEC tube formation by 7.2-fold compared to control [26]. Therefore we postulate that UPAR, Angiogenin, VEGF, IGF, Tie-2/TEK, IL-6 might be of certain significance for MVs in promoting angiogenesis at hypoxic conditions.

We select UPAR and Angiogenin for immunoblotting detection to validate the result of antibody array. The result of western blot is mainly in accordance with our antibody array result. We used GAPDH as an internal reference. And GAPDH was confirmed to be present in MVs by our immunoblotting result. GAPDH is a key enzyme in the ATP generating stage of glycolysis. Intracellular transfer of glycolytic enzymes by MVs could increase glycolytic flux to generate more ATP to provide energy for angiogenesis and enhance repair [27]. Our flow cytometric analysis showed an expression of MSC specific antigen CD73 in MVs. CD73 is an ecto-5′-nucleotidase and is the only enzyme known to hydrolyze extracellular AMP to adenosine. Adenosine is an activator of ERK and AKT phosphorylation and has been shown to mediate diverse cell protective, vasodilatory, and angiogenic responses [28].

We used an antibody array to analyze the expression of angiogenesis cytokines from MVs and parent MSCs and found upon serum-deprivation condition, MSCs secrete MVs that contain a variety of factors contributing to their proangiogenic function. Among those factors, angiogenin, VEGF, MCP-1, and VEGF R2 might be of greater importance than the other cytokines. UPAR, Angiogenin, VEGF, IGF, Tie-2/TEK, IL-6 might be responsible for hypoxia-augmented proangiogenic activity of MVs.
